# Clinical characteristics and computed tomography findings in Arab patients diagnosed with pulmonary sarcoidosis

**DOI:** 10.4103/0256-4947.57168

**Published:** 2009

**Authors:** Esam H. Alhamad, Mohammed O. Alanezi, Majdy M. Idrees, Mohammad K. Chaudhry, Ali M. AlShahrani, Arthur Isnani, Shaffi Shaikh

**Affiliations:** a From the Department of Medicine, King Saud University, Riyadh, Saudi Arabia; b From the Department of Medicine, King Abdulaziz Medical City, King Saud University, Riyadh, Saudi Arabia; c From the Department of Medicine, Riyadh Military Hospital, King Saud University, Riyadh, Saudi Arabia; d From the Department of Medicine, College of Medicine and Research Center, King Saud University, Riyadh, Saudi Arabia; e From the Department of Family & Community Medicine, King Saud University, Riyadh, Saudi Arabia

## Abstract

**BACKGROUND AND OBJECTIVE:**

Sarcoidosis is prevalent worldwide with significant heterogeneity across different ethnic groups. We aimed To describe the clinical characteristics and computed tomography findings among Arab patients with pulmonary sarcoidosis.

**METHODS:**

A retrospective study of patient demographics, symptoms, co-morbid illness, sarcoidosis stage, treatment, pulmonary function and CT results.

**RESULTS:**

Of 104 patients, most (77%) were 40 years of age or older at diagnosis, and females in this category (≥40 years) significantly outnumbered male patients (69/104 (66.3%) vs. 35/104 (33.7%), *P*=.003). The most common complaints were dyspnea (76%), cough (72.1%) and weight loss (32.7%). The majority of patients displayed impairment in lung function parameters at presentation. However, significant impairment in forced vital capacity, percentage predicted (FVC%) (<50%) was present in only 17% of patients. The most frequent CT finding was mediastinal lymph node enlargement in 49 patients (73.1%). Parenchymal abnormalities indicating lung fibrosis were noted in 31 patients (46.3%), and traction bronchiectasis was the most common (35.8%) fibrotic pattern detected on CT scans.

**CONCLUSION:**

At presentation, clinical manifestations of sarcoidosis among this sample of Arab patients were similar to reports from other nations. Further studies are needed to explore the effects of race and ethnicity on disease severity in the Middle East.

Sarcoidosis is a granulomatous disease of unknown etiology that most commonly affects the lungs and intrathoracic lymph nodes, but also has protean extrapulmonary manifestations. The disease is prevalent worldwide. However, significant heterogeneity in the frequency, clinical characteristics and severity of disease is observed across different ethnic groups and between the genders.^1^–^5^ Furthermore, radiographic findings at the time of diagnosis and the rate of radiographic clearing with treatment were also different among different ethnic groups.^5^ Limited reports on sarcoidosis in Arab patients have appeared,^6^–^8^ and to the best of our knowledge, no specific study has described CT findings among Arab patients diagnosed with pulmonary sarcoidosis. This retrospective study was conducted to explore the clinical, physiological, and radiological characteristics of pulmonary sarcoidosis in a sample of Arab patients.

## METHODS

We conducted a retrospective review of all patients diagnosed with biopsy-proven pulmonary sarcoidosis at three centers (King Khalid University Hospital, King Abdulaziz Medical City and Riyadh Military Hospital) in Riyadh, Saudi Arabia, between January 1992 and December 2007. Patients were identified from the records of outpatient pulmonary clinics and hospital medical departments. This investigation was approved by the ethics committee of each participating hospital. Sarcoidosis was diagnosed based on the latest American Thoracic Society (ATS), European Respiratory Society (ERS) and World Association of Sarcoidosis and Other Granulomatous Disorders (WASOG) criteria.^1^ Although these guidelines were published only in 1999, we verified that all patients included in the present study fulfilled all designated criteria. Patients displaying evidence of mycobacterial or fungal infection and those with a history of ingestion of drugs or agents causing granulomatous lung disease were excluded. Data included patient demographics, symptoms, details of co-morbid illnesses, sarcoidosis stage, treatment, pulmonary function and CT findings.

The modified Scadding^9^ classification system was applied to stage chest radiography (CXR) findings and patients were grouped as Stage 0 (no radiographic abnormality), Stage 1 (bilateral hilar adenopathy with no parenchymal abnormality), Stage 2 (bilateral hilar adenopathy with interstitial parenchymal infiltrates), Stage 3 (interstitial parenchymal infiltrates without hilar adenopathy) and Stage 4 (pulmonary fibrosis). CT reports collected from medical records were assessed for the presence of the following recognized CT patterns:^10^ (1) mediastinal and/or hilar lymph node enlargement, (2) ground-glass opacity, (3) consolidation, (4) nodules <3 cm in diameter, (5) thickening of bronchovascular bundles, (6) linear opacity including interlobular septal lines and interstitial thickening, (7) bronchial narrowing secondary to lymph node compression, (8) bullae, (9) and features indicating scarring and fibrosis (grouped together) that included the following: traction bronchiectasis, honeycombing, cysts, and/or volume loss. All CT scans were performed within 1 to 10 years after diagnosis (n=67). Extrapulmonary organ involvement was described according to the criteria established by Judson and colleagues.^11^ Treatment was defined as a requirement for corticosteroids with or without immunosuppressive therapy within 3 months of data collection. Treatment regimens applied were grouped as follows: none (no treatment), corticosteroids, corticosteroids and azathioprine, corticosteroids and methotrexate, and oxygen therapy. Treatment decisions were based on individual physician opinions. Disease duration was defined from the time of diagnosis until the last follow-up.

Spirometry was performed and plethysmographic lung volume and single-breath diffusion capacity of carbon monoxide (DLCO) measured. Forced vital capacity (FVC), forced expiratory volume in 1 sec (FEV_1_), and total lung capacity (TLC), were determined, and values were expressed as percentages of predicted values appropriate for gender, weight and age. Measures of DLCO were made by the single-breath technique and results were corrected for patient hemoglobin level. Calculation of lung volume and measurement of diffusion capacity were performed according to the recommendations of the American Thoracic Society.^12^,^13^ All lung function measurements used in this study were performed within 4 weeks of diagnosis (n=94 for FVC and FEV_1_; n=75 for TLC; and n=50 for DLCO).

Data were entered into MS Excel and analyzed using the statistical Software Package for the Social Sciences (SPSS pc+ Version 13.0; SPSS Inc, Chicago, IL). Descriptive statistics (mean, standard deviation and percentage) were applied to summarize quantitative and qualitative variables. The chi-square test was used to compare the proportion of qualitative variables. A *P* value <.05 was considered statistically significant.

## RESULTS

**Figure 1:**
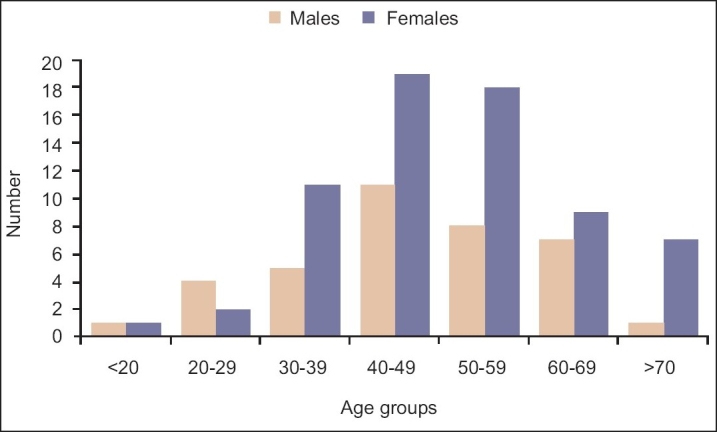
Frequency distribution of sarcoidosis by age group (males vs. females).

Our study cohort included 84 native Saudi (80.8%) patients and 20 (19.2%) patients of other Arab origins. The female-to-male ratio was 1.8:1 (Table 1). Mean (SD) age at diagnosis was 46.9 (12.5) years. Females tended to be older than males at the time of diagnosis 50.1 (13.8) *vs*. 48.1 (14.6) years (*P*=.5). The majority of patients (77%) were 40 years or older at diagnosis (Figure 1), and females in this category significantly outnumbered male patients (66.3% vs. 33.7%, *P*=.003). At the time of initial visit, the mean symptom duration was 8 months, with dyspnea as the most common complaint. The mean follow-up duration from the date of diagnosis was 5.4 years.

**Table 1:** Demographic and clinical characteristics of patients with sarcoidosis.

Variables	Mean	Standard Deviation	No. (%)
Age (years)	46.9	12.5	

Gender			
Males			37 (35.6)
Females			67 (64.4)

Male: Female ratio			1: 1.8

Smoker			16 (15.4)

Non-smoker			88 (84.6)

Presenting symptoms			
Dyspnea			79 (76.0)
Cough			75 (72.1)
Weight loss			34 (32.7)

Co-morbid illnesses			
Diabetes mellitus			33 (31.7)
Hypertension			26 (25.0)
Ischemic heart disease			7 (6.7)

Chest radiographic stage			
Stage 0			6 (5.8)
Stage I			24 (23.1)
Stage II			44 (42.3)
Stage III			17 (16.3)
Stage IV			13 (12.5)

Extra-pulmonary organ involvement			
Eye			10 (9.6)
Liver, spleen			9 (8.6)
Skin			4 (3.8)
Bone			3 (2.9)
Kidneys			2 (1.9)
Hypercalcemia			8 (7.9)
Other*			3 (2.9)

Treatment			
None			32 (30.8)
Corticosteroids			54 (52)
Corticosteroids + Azathioprine			8 (7.7)
Corticosteroids + Methotrexate			7 (6.7)
Oxygen therapy			3 (2.9)

* Central nervous system, n=1; heart, n=1; muscle, n=1

The procedures performed to obtain diagnostic histological specimens included bronchoscopy (trans-bronchial [TBB] or bronchial biopsy) in 57 patients (54.8%), mediastinoscopy in 14 (13.5%), surgical lung biopsy in 10 (9.6%), and other biopsies (peripheral lymph node, liver, skin, or renal) in 23 (22.1%) patients. The majority of patients displayed impairment in lung function parameters at presentation. The mean (SD) for FVC, FEV_1_, TLC, and DLCO as percentages of predicted values were 74.7 (22.4), 74.5 (21.6), 78.1(18.9) and 61.9 (19.7), respectively. Significant impairment in FVC of <50% was present in only 17% of patients. Approximately two-thirds of patients continued to receive corticosteroids and/or immunosuppressive medication following examination of their previous medical records.

The most frequent CT patterns observed were mediastinal lymph node enlargement in 49 (73.1%) patients (Table 2). Parenchymal abnormalities indicating fibrosis were noted in 31 patients (46.3%) and the most common fibrotic pattern was traction bronchiectasis. Four patients died during the study period mean follow-up 5.4 years, including three females. In all cases, death was attributed to respiratory failure.

**Table 2:** CT findings in 67 patients with pulmonary sarcoidosis.

CT scan features	n	%
Mediastinal lymph node enlargement Hilar lymph node enlargement	49	73.1
Unilateral	8	11.9
Bilateral	28	41.8
Nodules	36	53.7
Traction bronchiectasis	24	35.8
Ground glass opacities	22	32.8
Thickening of bronchovascular bundles	15	22.4
Interlobar lines	12	17.9
Cysts	9	13.4
Septal lines	8	11.9
Honeycombing	7	10.4
Consolidations	4	6.0
Bronchial narrowing secondary to lymph node compression	4	6.0
Bullae	1	1.5

## DISCUSSION

Sarcoidosis affects individuals worldwide, and shows a predilection for adults less than 40 years of age.^1^ However, several investigators have identified older patients, with women tending to be of greater age than men.^4^,^14^ In the large “case control etiologic study of sarcoidosis” (ACCESS) study involving several geographic regions of the United States, women patients were generally older than men and one-third of patients were 60 years of age or over.^2^ In the present analysis, mean patient age at diagnosis was 49.3 years, consistent with earlier reports on Arab patients with sarcoidosis.^6^,^8^

A bimodal incidence peak, mostly in women, has been described in Nordic countries and Japan, with the second peak occurring at an average age of 50 years or older.^4^,^14^ In our study, the peak occurred at 41-50 years of age in males and 41-60 years in females, with a higher proportion of women patients above the age of 70 years. Limited reports appearing to date have described sarcoidosis among elderly patients.^15^–^17^ In an earlier study, Chevalet and colleagues reviewed 30 elderly patients (21 women and 9 men) above 70 years of age (mean age, 74 years) diagnosed with sarcoidosis.^15^ Whereas alterations in general health were the major diagnostic sign, overall prognosis was similar to that in young subjects. Interestingly, in our cohort, the mean age of elderly patients diagnosed after 70 years of age was higher at 79.6 years (73-90 years), with a preponderance of women. Intrathoracic involvement, along with symptoms of cough and dyspnea, were the predominant findings. As elderly patients additionally present with characteristic symptoms of pulmonary and multisystem disease, sarcoidosis should be considered in the differential diagnosis of these disorders.

In the current cohort, 64.4% patients were women, consistent with previous studies including reports on Arab patients with sarcoidosis, disclosing a higher disease rate in women compared to men.^4^–^6^,^8^ Pulmonary manifestations are typically predominant, but virtually any organ system can be involved.^1^ The coughing frequency at the time of diagnosis varies among regions, from 3% of patients in Japan^5^ to 100% in another series.^18^ Moreover, the mode of presentation differs between countries. For instance, Japanese patients are more likely to present with ocular symptoms, whereas respiratory symptoms and erythema nodosum are the common clinical presentations in Finland.^5^ In our patients, dyspnea and cough were the most common complaints, in keeping with previous reports on sarcoidosis in Arab populations.^6^,^8^

The prevalence and distribution of extrapulmonary involvement varies worldwide. These variations can be explained by varying degrees of susceptibility according to race and ethnicity, and are probably related to moderation of the effects of genetic background. The rate of extrapulmonary involvement ranged from 1% to 9.6% in our patient population, depending on the organ involved. However, we believe that our data, along with those of earlier studies focusing on Middle Eastern populations,^6^,^8^ do not represent an accurate estimation of extrapulmonary organ involvement, that would permit meaningful comparison with findings reported elsewhere, because our patients were recruited from pulmonary clinics. Consequently, individuals presenting to other specialists would not have come to our attention.

Chest radiographic findings appear to vary worldwide. For example, higher chest radiographic stages are detected among Finnish patients, whereas Japanese patients are more likely to present with Stage I disease.^5^ In our study, 42.3% of the cohort presented with Stage II disease, in keeping with other reports on Middle Eastern patients with sarcoidosis.^6^–^8^ At the time of diagnosis, the percentage of sarcoidosis patients diagnosed with radiographic Stage 0 varied significantly (5.7% in the current study, 1% in Finnish patients, and 19% in Japanese patients).^5^ The number of patients with radiographic Stage IV disease was higher in the present study (12.5%) compared to what was reported by Behbehani and colleagues (1.8%).^6^ Thus, race and/or ethnicity may contribute to susceptibility to sarcoidosis and disease severity.

Aberrations in pulmonary function tests (PFTs) are common in patients with sarcoidosis, with abnormalities detected in 20% of patients with Stage 1 disease and in 40% to more than 70% of patients with Stage 2, 3, or 4 disease.^1^ The majority of our cohort exhibited abnormalities in PFTs, with 17% of patients displaying markedly reduced FVC, <50% of normal, at presentation. Given the limited predictive value of baseline PFTs^19^–^24^ and the variable natural history of the disease, other outcome measures that provide better objective information, and thus afford a more accurate predictive model for morbidity and mortality, are essential.

Several studies have investigated the role of CT, including high resolution CT (HRCT), in sarcoidosis patients.^25^–^30^ However, routine CT scanning is not advocated in management of sarcoidosis except in patients in whom normal or atypical chest radiography is evident, or specific complications are suspected, such as in patients with pulmonary fibrosis, bronchiectasis, aspergilloma, or malignancy.^1^,^31^ In the present study, mediastinal lymph node enlargement was the most common CT finding, in keeping with previous reports on patients with pulmonary sarcoidosis.^25^,^28^ Correlation of HRCT findings with pulmonary function studies has been variable, probably related to interstitial vs. bronchial involvement, or reflecting different HRCT scoring applied in various reports.^27^–^29^ In our current study we did not attempt to determine such relationships, as CT scans were not obtained systematically, and were not performed simultaneously with PFT evaluation. Abehsera and associates^30^ evaluated chest CT scans of sarcoid patients who displayed chest radiographic evidence of fibrotic changes (Stage IV disease), and found the following three different CT patterns: bronchial distortion including traction bronchiectasis (predominantly central in nature), honeycombing (predominantly peripheral and often in the upper zones), and a linear pattern (predominantly diffuse in nature). These patterns were recognized in 47%, 29%, and 24% of patients, respectively. Notably, almost half of our patients had developed pulmonary fibrosis based on CT, where traction bronchiectasis was the most frequent CT pattern found, consistent with data of a previous study.^30^ Identification of prognostic variables based on HRCT patterns has also produced discordant results,^27^,^30^,^32^,^33^ probably related to differences in the methodology used in various studies or (perhaps more likely) related to disease expression moderated by gene-environmental interaction. Further large-scale studies are warranted to explore such effects within the Arab population.

Our study had several limitations. First, this was a retrospective review, and several values, such as TLC and DLCO, were not available for all patients. Second, there was a selection bias, because the study was performed in tertiary hospitals, in which patients with severe complaints and in advanced stages of sarcoidosis were more likely to be referred.

In conclusion, a significant number of this sample of Arab patients diagnosed with pulmonary sarcoidosis were symptomatic and displayed impairment in lung function parameters at presentation. Mediastinal lymphadenopathy was the most frequent CT finding and a significant number of patients progressed to pulmonary fibrosis.

Future studies need to focus on disease prevalence and patterns preceding development of lung fibrosis attributable to sarcoidosis.
